# Modelling premature cardiac aging with induced pluripotent stem cells from a hutchinson-gilford Progeria Syndrome patient

**DOI:** 10.3389/fphys.2022.1007418

**Published:** 2022-11-23

**Authors:** Gustavo Monnerat, Tais Hanae Kasai-Brunswick, Karina Dutra Asensi, Danubia Silva dos Santos, Raiana Andrade Quintanilha Barbosa, Fernanda Cristina Paccola Mesquita, Joao Paulo Calvancanti Albuquerque, Pires Ferreira Raphaela, Camila Wendt, Kildare Miranda, Gilberto Barbosa Domont, Fábio César Sousa Nogueira, Adriana Bastos Carvalho, Antonio Carlos Campos de Carvalho

**Affiliations:** ^1^ Institute of Biophysics Carlos Chagas Filho, Federal University of Rio de Janeiro, Rio de Janeiro, Brazil; ^2^ Laboratory of Proteomics, LADETEC, Institute of Chemistry, Federal University of Rio de Janeiro, Rio de Janeiro, Brazil; ^3^ National Center of Structural Biology and Bioimaging, CENABIO, Federal University of Rio de Janeiro, Rio de Janeiro, Brazil; ^4^ Proteomic Unit, Institute of Chemistry, Federal University of Rio de Janeiro, Rio de Janeiro, Brazil; ^5^ National Science and Technology Institute in Regenerative Medicine, Rio de Janeiro, Brazil

**Keywords:** aging, progeria, proteomics, pluripotent stem cell (PSC), cardiology, metabolism, mass spectrometery (MS)

## Abstract

Hutchinson-Gilford Progeria Syndrome (HGPS) is a rare genetic disorder that causes accelerated aging and a high risk of cardiovascular complications. However, the underlying mechanisms of cardiac complications of this syndrome are not fully understood. This study modeled HGPS using cardiomyocytes (CM) derived from induced pluripotent stem cells (iPSC) derived from a patient with HGPS and characterized the biophysical, morphological, and molecular changes found in these CM compared to CM derived from a healthy donor. Electrophysiological recordings suggest that the HGPS-CM was functional and had normal electrophysiological properties. Electron tomography showed nuclear morphology alteration, and the 3D reconstruction of electron tomography images suggests structural abnormalities in HGPS-CM mitochondria, however, there was no difference in mitochondrial content as measured by Mitotracker. Immunofluorescence indicates nuclear morphological alteration and confirms the presence of Troponin T. Telomere length was measured using qRT-PCR, and no difference was found in the CM from HGPS when compared to the control. Proteomic analysis was carried out in a high-resolution system using Liquid Chromatography Tandem Mass Spectrometry (LC-MS/MS). The proteomics data show distinct group separations and protein expression differences between HGPS and control-CM, highlighting changes in ribosomal, TCA cycle, and amino acid biosynthesis, among other modifications. Our findings show that iPSC-derived cardiomyocytes from a Progeria Syndrome patient have significant changes in mitochondrial morphology and protein expression, implying novel mechanisms underlying premature cardiac aging.

## 1 Introduction

Hutchinson-Gilford Progeria Syndrome (HGPS) is an extremely rare genetic disorder. Patients with HGPS present severe cardiovascular complications associated with a premature process of aging (Hennekam, 2006; Capell et al., 2008; Merideth et al., 2008). At the genetic level, HGPS is caused by a single mutation in the Lamin A (LMNA) gene, leading to the expression of a mutant protein isoform. The mutated protein, also known as Progerin (isoform 6), is accumulated in the cells, promoting several effects, such as nuclear blebbing and stiffness, mitochondrial dysfunction, and epigenomic alterations, leading to disturbed protein homeostasis and accelerated senescence (Capell et al., 2007; Merideth et al., 2008; Olive et al., 2010; Liu et al., 2011; Bonello-Palot et al., 2014; Gordon et al., 2018; Hamczyk et al., 2018).

The phenomenon of aging is a biological process of progressive accumulation of physiologic changes, decreasing the ability to maintain the body’s homeostasis (Kenyon, 2010). Aging is not a disease, but it dramatically increases the risk of developing chronic cardiovascular (North and Sinclair, 2012) and metabolic diseases (Barzilai et al., 2012; Bonomini et al., 2015). The aging process varies greatly among organisms, differing more than 100-fold among species (Ma et al., 2015), but the reasons for this large variation are not fully understood. Initially, one of the main mechanisms suggested as responsible for aging was DNA damage accumulation associated with decreased activity of telomerase (Harley et al., 1990). Currently, additional mechanisms are discussed, such as mitochondrial dysfunction and ROS generation (Chen et al., 2017), metabolic alterations (Monnerat et al., 2018b) and alterations in protein expression of key pathways, which could regulate aging (Rando, 2006; Kenyon, 2010; López-Otín et al., 2013).

Investigation of cardiac diseases at the cellular and molecular level is complicated due to the invasiveness of obtaining cardiac tissue samples and maintaining functional primary cardiac cells under culture conditions. Nowadays, however, functional cardiomyocytes (CM) can be obtained with *in vitro* based on cell reprogramming using induced pluripotent stem cells (iPSC) (Takahashi et al., 2007; Zhang et al., 2009). The aim of the present study was to generate CM from an HGPS patient using iPSCs in order to investigate the underlying cellular and molecular mechanisms of cardiac premature aging.

## 2 Materials and methods

### 2.1 Cell culture and cardiomyocyte differentiation

**FIGURE 1:**
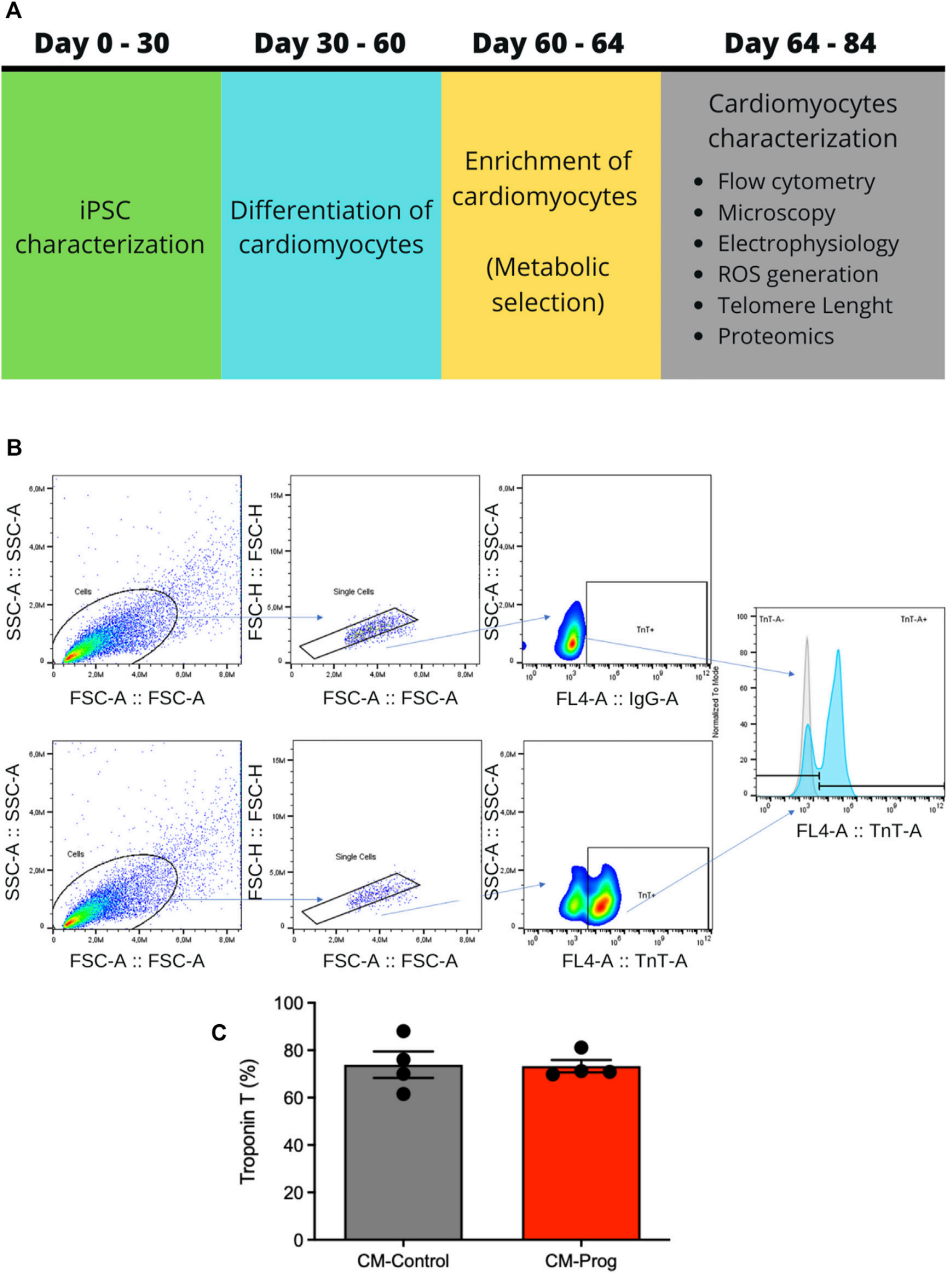
Study design and iPSC-derived cardiomyocyte generation **(A)** Schematic representation of the study design. **(B)** Gate strategy is shown in flow cytometry analysis of Troponin T (TnT) expression on cardiomyocytes derived from iPS cells. First, cells were gated by the scattering light pattern (forward scatter–FSC and side scatter–SSC), then single cells were selected and finally, the intensity of fluorescence was determined by the secondary antibody staining. Cardiomyocytes stained only with secondary antibody (Alexa-Fluor 647). Cardiomyocytes stained with anti-TnT primary antibody followed by secondary antibody staining (Alexa-Fluor 647). Histogram overlap of secondary-stained (grey curve) and TnT-stained cells (blue curve). The *x*-axis shows the intensity of fluorescence for TnT. **(C)** The efficiency of cardiomyocyte generation in consecutive paired cellular differentiations. The columns represent the percentage of cardiac troponin T positive cells after differentiation, and the bars represent SEM, and non-parametric Mann-Whitney test was applied (CM-Control n = 4; CM-Prog *n* = 4).

Induced pluripotent stem cells derived cardiomyocytes were generated as summarized in Figure 1A. Two iPSC lineages (we used four independent clones for each cell line investigated) were used in this work, one derived from a healthy donor previously described by our group (Mesquita et al., 2015), used as control, and the other, obtained from The Progeria Research Foundation/Cell and Tissue Bank, derived from a patient with HPGS. The experimental protocol was approved by the research ethics committee (IRB) of the National Institute of Cardiology under number 24138414.1.0000.5272. The iPSC from the Progeria patient presented the classical HGPS LMNA Exon 11, heterozygous c.1824C>T (p.Gly608Gly) mutation (PRF-Cell line: HGADFN167 iPS1J that was previously characterized (Atchison et al., 2020). The mutation was confirmed in the cardiomyocytes by Sanger sequencing (Supplementary Figure S1). The iPSCs were cultivated under feeder-free conditions using mTeSR™1 medium (cat. 85851, STEMCELL Technologies, Vancouver, BC, Canada). For cardiac differentiation, the iPSCs were plated at high-density (3 × 10^5^ iPSCs/well) for 72 h in 48-well culture plates covered with an hESC-qualified BDMatrigel™ matrix (cat. no. 354277, BD Biosciences, San Jose, CA, United States ) diluted 1:100 in PBS. On day 0, iPSCs were treated with 12 µM of GSK3 inhibitor, CHIR99021 (cat. 4,423, Tocris, Minneapolis, MN, United States ) in RPMI 1640 medium (cat.22400-021, ThermoFisher Scientific, Waltham, MA, United States ) with B-27 minus insulin supplement (cat. A189561, ThermoFischer Scientific, Waltham, MA, United States ) for 24 h. During days 1 and 2, the iPSCs were cultivated with RPMI 1640 medium supplemented with B-27 minus insulin. To achieve cardiac mesoderm specification, on day 3, 10 µM of XAV 939 (cat. 3,748, Tocris, Minneapolis, MN, United States ) were added to the culture medium (RPMI + B-27 minus insulin) and on day 4 this medium was replaced by fresh medium with 5 µM of XAV 939. RPMI + B-27 minus insulin medium was replaced daily on days 5 and 6. On day 7, RPMI was supplemented with B-27 with insulin (cat. 17504044, ThermoFischer Scientific, Waltham, MA, United States ) and spontaneous beating cells were observed. The RPMI medium supplemented with B-27 with insulin was replaced daily until day 10. After day 10, the medium was replaced twice a week until day 30, followed by a 4-day duration metabolic selection to enrich cultures in cardiomyocytes. Cultured cells were treated with 4 mM lactate (cat. 71718-10g, Sigma Aldrich, St. Louis, MO, United States ) diluted in DMEM no glucose (cat. 11966-025, ThermoFischer Scientific, Waltham, MA, United States ). In order to evaluate the efficiency of cardiomyocyte differentiation and enrichment, cardiac Troponin-T positive cells were quantified by flow cytometry after the metabolic selection. Cardiomyocytes were detached from cell plates using Trypsin-EDTA (cat. 25200-72, Gibco, ThermoFischer Scientific, Waltham, MA, United States ). The cells were then fixed with 4% formaldehyde (cat. 158127-300, Sigma-Aldrich, St. Louis, MO, United States ) for 20 min at room temperature followed by membrane permeabilization with 0.3% Triton X-100 (cat.9284, Sigma Aldrich, St. Louis, MO, United States ) for 20 min at room temperature. The cells were then stained for 30 min at 4°C with anti-Troponin T cardiac isoform antibody (cat. MS-295-P1, ThermoFischer Scientific, Waltham, MA, United States ) diluted 1:100 in buffer solution (PBS with 0.5% BSA). After washing, the cells were stained for 20 min at 4°C, in the dark, with Alexa Fluor 647 goat anti-mouse IgG (H + L) secondary antibody (cat. A- 21236, ThermoFisher Scientific, Waltham, MA, United States ) diluted 1:400 in buffer solution. The cells stained only with secondary antibody were used as unspecific stained control. The acquisition was performed in a BD Accuri C6 flow cytometer (BD Biosciences, San Jose, CA, United States ) and the data were analyzed by FlowJo software version X.1. Only cultures with more than 70% cardiomyocytes were used to perform the experiments.

### 2.2 Electrophysiology and action potential recordings

35-mm plates with 1 × 105 iPSCs derived cardiomyocytes were transferred to a recording chamber as described previously (Silva Dos Santos et al., 2018). Action potentials were recorded as described elsewhere (José et al., 2017). CM preparations were superfused with Tyrode’s solution containing (in mM): 150.8 NaCl, 5.4 KCl, 1.8 CaCl2, 1.0 MgCl2, 11.0 d-glucose, 10.0 HEPES (pH 7.4 adjusted with NaOH) saturated with oxygen at a perfusion flow rate of 0.5 ml/min (Miniplus 3, Gilson, Middleton, WI, United States ) and 37.0 ± 0.5 °C using Temperature Controller (Harvard Apparatus, Holliston, MA, United States ). Transmembrane potential was recorded using glass microelectrodes (40–80 MΩ DC resistance) filled with 2.7 M KCl connected to a Microelectrode Amplifier (MultiClamp 700B, Molecular Devices, San Jose, CA, United States ). Amplified signals were digitized (1,440 digidata A/D interface, Axon Instruments, Molecular Devices, San Jose, CA, United States ) and stored in a computer for later analysis using LabChart 7.3 software (ADInstruments, Bella Vista, NSW, Australia). The following parameters were analyzed: resting potential, maximum upstroke velocity, and action potential duration at 90% (APD90) repolarization, from three consecutive action potentials from each cell.

### 2.3 Electron tomography and 3D reconstruction

Cells were washed in Dulbecco’s PBS, pH 7.2, fixed for 1 h in a solution containing 2.5% glutaraldehyde, 4% freshly prepared formaldehyde in 0.1 M Sodium cacodylate buffer. Cells were then washed in the same buffer and postfixed in 1% OsO_4_ plus 0.8% ferrocyanide and 5 mM CaCl_2_ in 0.1 M cacodylate buffer, pH 7.2, for 1 h, dehydrated in ascending concentrations of ethanol and embedded in Polybed 812 epoxide resin. Two hundred-nanometer ribbons of serial sections of iPSCs derived CM were collected on Formvar-coated slot copper grids. Samples were poststained with uranyl acetate and lead citrate and incubated with 10 nm colloidal gold on both sides for 5 min and washed in distilled water as described previously (Wendt et al., 2016). Sections were observed in a 200 kV FEI Tecnai G2 transmission electron microscope (Tecnai G2, FEI Company, Eindhoven, Netherlands) equipped with a 4k CCD camera (Eagle, FEI Company, Eindhoven, Netherlands). Tilt series were acquired using Xplore 3D (FEI Company, Eindhoven, Netherlands). Tomograms were recorded between - 65 and +65 with an angular increment of 1°. Alignments were applied using fiducial markers and weighted back projections with the MOD software package. The IMOD package was used for segmentation and data analysis. Mitochondrial volume and the number of cristae were calculated from a total of 48 mitochondria (CM-Control n = 19; CM-Prog n = 29) in tomographic reconstructions obtained from at least six fields, assuming a sample thickness of 200 nm. Representative images for nucleus morphology were also obtained in the same prepared samples. Three cells were analyzed in control and in Progeria cardiomyocytes from different differentiations.

### 2.4 Immunofluorescence

The cells were fixed with 4% paraformaldehyde for 20 min at room temperature. Then they were washed three times with PBS 1x for 5 min. Next, the cells were permeabilized with 0.3% Triton diluted in 1x PBS for 15 min. Subsequently, blocking was done using a 5% BSA solution diluted in PBS 1x for 60 min. We then placed the primary antibody in question in PBS-BSA 3% at 1:100 (LMNA, cat. MA3-1,000, ThermoFisher scientific) for Lamin A/C; and 1:200 (Troponin T, cat. MS-295-P1, ThermoFisher scientific) for Troponin T, cardiac isoform, dilutions, and incubated overnight at 4°C. The following day, we washed 3x with 1x PBS for 5 min. After the washes, we placed the secondary antibody Cy™3 AffiniPure Donkey Anti-Mouse IgG (H + L) (cat. 715150, Jackson ImmunoResearch) diluted in PBS-BSA 3% at a 1:400 ratio for 2 h at room temperature. After incubation, we washed 3x with 1x PBS for 5 min. Finally, we placed DAPI (cat. D9542, Sigma-Aldrich) for 5 min and washed 3x times with PBS 1x for 5 min. The coverslips were sealed using 13 µL of Fluoroumont (ThermoFisher scientific). Images were taken with the ×100 objective on the Elyra PS.1 confocal microscope (Carl Zeiss), ZEN 2012 SP5 software (Carl Zeiss), at the Advanced Microscopy Unit (UMA) of the National Center for Structural Biology and Bioimaging (UFRJ, Rio de Janeiro).

### 2.5 Telomere length by quantitative PCR (qPCR)

DNeasy Blood & Tissue Kit (Qiagen, Venlo, Netherlands) was used in DNA extraction according to the manufacturer’s instructions. The genomic DNA integrity was verified by electrophoresis using 50 ng of DNA in 1.5% agarose gel at 200 V for 45 min. DNA samples were frozen at -20 °C until qPCR analysis. Telomere length measurement was based on the protocol previously described (Gutierrez-Rodrigues et al., 2014). Briefly, qPCR was performed in 24 µL final volume reactions including 1.6 ng of genomic DNA, 2x RotorGene SYBR Green PCR Master Mix (Qiagen, Venlo, Netherlands), 300 nM of primer Tel forward (CGG​TTT​GTT​TGG​GTT​TGG​GTT​TGG​GTT​TGG​GTT​TGG​GTT) and 300 nM of primerTel reverse (GGC​TTG​CCT​TAC​CCT​TAC​CCT​TAC​CCT​TAC​CCT​TAC​CCT) or 300 nM of primer 36B4 single gene forward (CAG​CAA​GTG​GGA​AGG​TGT​AAT​CC) and 500 nM of primer 36B4 single gene reverse (CCC​ATT​CTA​TCA​TCA​ACG​GGT​ACA​A).

Amplification cycling was conducted in Step One System (Qiagen, Venlo, Netherlands) as follow: 5 min at 95°C followed by 25 cycles of 7 s at 98°C and 10 s at 60°C for telomere reactions or 5 min at 95 °C followed by 35 cycles of 7 s at 98°C and 10 s at 58°C for single gene reactions. Telomere length (*x*) was calculated as telomere to single copy gene ratio (T/S ratio) and based on the calculation of the ΔC*t* [C*t* (telomeres)/C*t* (single gene)]. Telomere length was expressed as the relative T/S ratio, which was normalized to the average T/S ratio of reference sample [2−(ΔC*tx* − ΔC*tr*) = 2^−ΔΔCT^]. The linear regression equation used to convert T/S ratio values in kilobases was: telomere length (kb) = 4.330x+5.07, based on the correlation with Southern blot analyses, where x corresponds to the T/S ratio value.

### 2.6 Intracellular ROS by DCF assay

The cells were dissociated with TrypLE™ express (Gibco, ThermoFischer Scientific, Waltham, MA, United States ) and 1 × 10^5^ control or HGPS cells (iPSC or CM) were incubated with 10 µM of CM-H2DCFDA (cat. C6827, Invitrogen, Carlsbad, CA, United States ) for 30 min at 37°C. The samples were centrifuged at 4°C, resuspended in PBS and fluorescence was immediately measured with excitation and emission wavelengths of 490 and 525 nm, respectively, in Victor™ X5 microplate reader to determine intracellular generation of ROS. The results were expressed as Mean Fluorescence intensity per 1 × 10^5^ cells. Unstained cells were used for determining autofluorescence.

### 2.7 Mitochondria staining by mitotracker assay

The cells were dissociated with TrypLE™ express (Gibco, ThermoFischer Scientific, Waltham, MA, United States ) and 1 × 10^5^ control or HGPS cells (iPSC or CM) were incubated with 0.5 µM of MitoTracker^®^ Red FM (cat. M22425, Invitrogen, Carlsbad, CA, United States ) for 30 min at 37 °C. The samples were centrifuged at 4 °C, resuspended in PBS and fluorescence was immediately measured with excitation and emission wavelengths of 581 and 644 nm, respectively, in Victor™ X5 microplate reader (PerkinElmer, Waltham, MA, United States ) to indirectly determine mitochondria content. The results were expressed as Mean Fluorescence intensity per 1 × 10^5^ cells. Unstained cells were used for determining autofluorescence.

### 2.8 Proteomics

#### 2.8.1 Sample preparation

Briefly, protein samples from iPSCs derived cardiomyocytes were precipitated with 10% (1:4 v/v) trichloroacetic acid (Sigma-Aldrich, St. Louis, MO, United States ) in acetone (Sigma-Aldrich, St. Louis, MO, United States ) by incubation on ice overnight followed by centrifugation for 15 min at 4°C and 15,000 rpm. Samples were washed three times with cold acetone and air dried. Proteins were suspended in 15 μL of 7 M urea/2 M thiourea (Sigma-Aldrich, St. Louis, MO, United States ) and were quantified using a Quibit Protein Assay Kit (Thermo Scientific, Thermo Scientific, Waltham, MA, United States ). Proteins were reduced with 10 mM dithiothreitol (DTT; Sigma-Aldrich, St. Louis, MO, United States ) by incubation for 1 h at 30 °C followed by alkylation by incubation with 55 mM iodoacetamide (IAM; Sigma-Aldrich, St. Louis, MO, United States ) for 30 min in the dark at room temperature. After alkylation, 50 mM NH4HCO3 (10:1 v/v; Sigma-Aldrich, St. Louis, MO, United States ) and mass spectrometry-grade trypsin (Promega, Madison, WI, United States ) at a ratio of 50:1 (protein:trypsin) were added to the proteins, and were incubated overnight at 35°C. After digestion, the samples were acidified using 0.1% trifluoroacetic acid (TFA; Sigma-Aldrich, St. Louis, MO, United States ). Peptides were cleaned with an in-house prepared C-18 column and eluted in 50 μL of 50% acetonitrile (ACN)/0.1% TFA followed by 50 μL of 70% ACN/0.1% TFA, dried in a SpeedVac concentrator (Thermo Scientific, Waltham, MA, United States ) and resuspended in 15 μL of 0.1% formic acid (Sigma-Aldrich, St. Louis, MO, United States ). Peptides were quantified using a Quibit Protein Assay Kit and suspended to a final concentration of 0.25 μg/μL in 0.1% formic acid.

### 2.9 Sample and data analysis

Samples were analyzed in three technical replicates by liquid chromatography-tandem mass spectrometry (LC-MS/MS). Briefly, 4 μL of the diluted samples were applied to an EASY-nLC 1,000 system (Thermo Scientific, Waltham, MA, United States ) coupled online to an nESI-Q-Exactive Plus mass spectrometer (Thermo Scientific, Waltham, MA, United States ). Peptides were loaded into a trap column (EASY-ColumnTM, 2cm, ID100μm, 5um, 120A, C18-A1 -Thermo Scientific, Waltham, MA, United States ) and eluted in an analytical column (75 μm × 25 cm) packed in-house with ReproSil-Pur 120 C18-AQ, 3 µm (Dr. Maisch, Ammerbuch, Germany). Peptide separations were performed using a gradient from 95% solution A (0.1% formic acid, 5% acetonitrile) to 5–20% solution B (0.1% formic acid, 95% acetonitrile; Sigma-Aldrich, St. Louis, MO, United States ) over 120 min followed by 20–40% solution B over 40 min, followed by 40–95% solution B over 7 min and were maintained in 95% solution B for 13 min. MS1 spectra were acquired in a positive mode using the data-dependent automatic (DDA). Each DDA consisted of a survey scan in the m/z range of 350–2000 and a resolution of 70,000 (at m/z 200) with automatic gain control (AGC) target value of 1 × 10–6 ions. The 20 most intense ions were subjected to MS2 acquisition using HCD normalized dissociation (HCD) of previously selected ions, a resolution of 17,500 and AGC of 1 × 10–6 ions.

MS data were analyzed with Proteome Discoverer (version 2.1.0.81, Thermo Scientific, Waltham, MA, United States ) using a Uniprot *Homo sapiens* database and Sequest HT algorithm. Search parameters were for tryptic peptides, two missed cleavages, oxidation of methionine, n-terminal protein acetylation, as variable modification, and carbamidomethylation as static modifications, and a precursor mass tolerance of 10 ppm and fragment mass tolerance of 0.05 Da. A cutoff score was established to accept a false discovery rate (FDR) of 1%, using Percolator. Proteins were grouped according to the maximum parsimony approach. To be considered as identified, the protein had to be identified in at least in three LC-MS/MS runs per group. The identified proteins were submitted to the Gene Ontology Biological Process in the STRING online database, and biological processes and protein map interaction was carried out (Szklarczyk et al., 2017). Label free quantification was performed as previously described (Nogueira et al., 2018). Multivariate analysis was performed using principal component analysis (PCA) and univariate statistics with volcano plot considering FDR<0.1, as previously described (Roumeliotis et al., 2017), with Perseus software platform (Tyanova et al., 2016).

The mass spectrometry proteomics data have been deposited to the ProteomeXchange Consortium *via* the PRIDE (Perez-Riverol et al., 2022) partner repository with the dataset identifier PXD036557.

### 2.10 Statistical analysis

The data are shown as mean values ±SEM. Shapiro-Wilk test normality test was performed in the data. Multiple comparisons between groups were performed using Student’s t-test and non-parametric Mann-Whitney test.

Values of *p* < 0.05 were considered statistically significant. The statistics performed for proteomics is described above. All analyses were made using GraphPad Prism 9.0 (GraphPad Software, San Diego, CA, United States ).

## 3 Results

### 3.1 Efficiency of cardiomyocyte differentiation

The iPSCs were differentiated into CM as summarized in Figure 1A. In order to evaluate CM differentiation efficiency, the expression of Troponin-T was quantified. Figure 1B shows the gate strategy in flow cytometry analysis of Troponin T (TnT) expression on cardiomyocytes derived from iPS cells. As shown in Figure 1C, our method was able to generate an average of 80% CM and there were no differences in differentiation efficiency after metabolic selection (see Methods) between CM derived from the healthy donor (CM-Control) and from the HGPS patient (CM-Prog) in three independent paired cellular differentiation protocols. Troponin-T staining confirmed the presence of troponin-T in the generated cardiomyocytes (Supplementary Figure S2).

#### 3.2 Nuclear morphology of cardiomyocytes derived from HGPS

**FIGURE 2:**
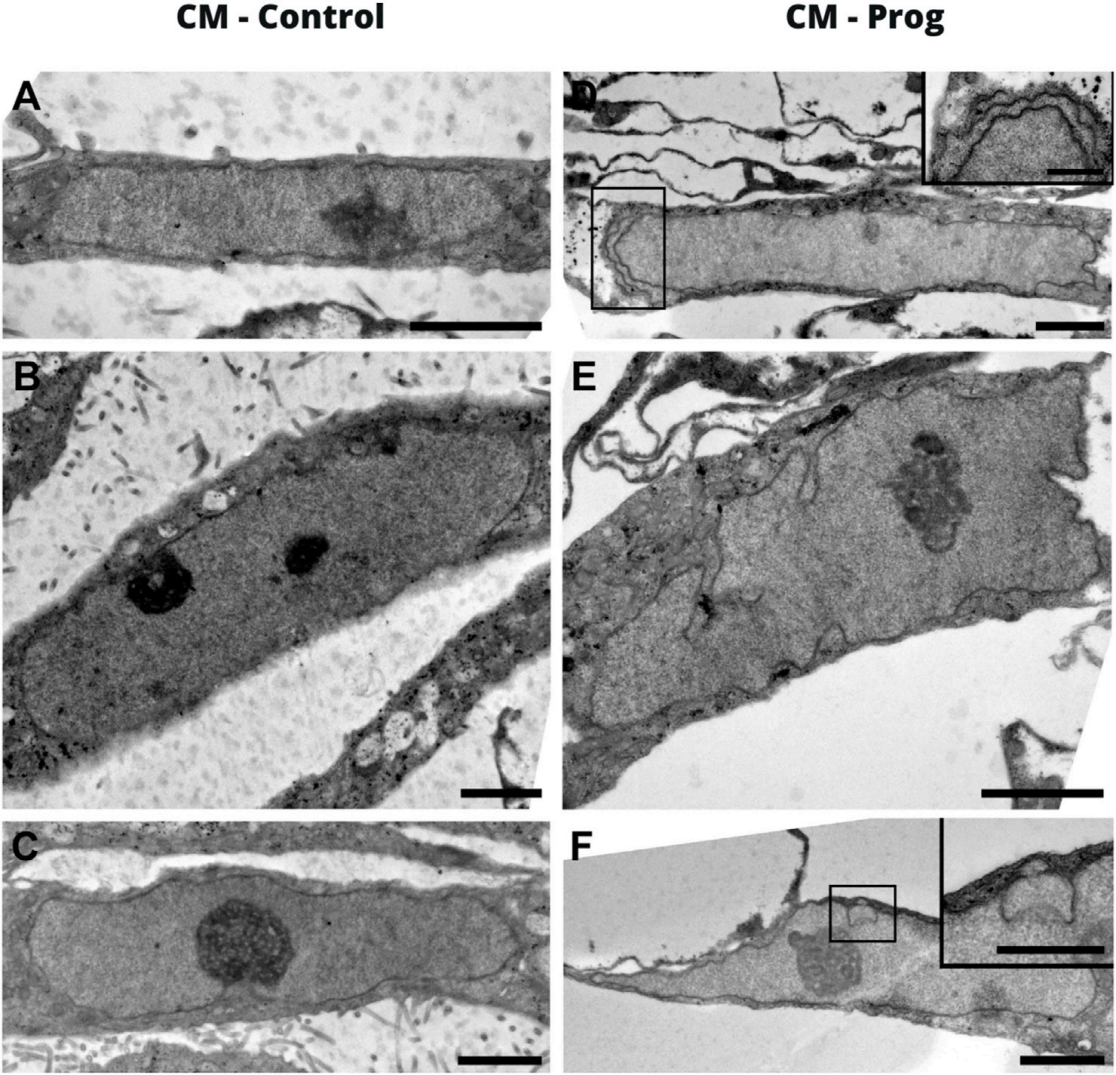
Nuclear morphology of iPSC-derived cardiomyocytes by electron tomography. **(A–C)** Images correspond to the CM-Control, and **(D–F)** to the CM-Prog. The scale bar on the images is 2 um and in the inserts it is 1 um.

A hallmark of Progeria syndrome is the nuclear morphology alteration in somatic cells. Aiming to evaluate if cardiomyocytes derived from HGPS, an electro tomography imaging model was applied for morphological evaluation. As illustrated with a representative image in Figure 2, the CM generated from a healthy donor presented a normal nuclear morphology (Figures 2A–C). However, the CM derived from the HGPS patient presented a clear morphological abnormality (Figures 2D–F). Furthermore, these results were further confirmed with confocal microscopy using DAPI and Lamin A/C staining (Supplementary Figure S3).

### 3.3 Functional cardiomyocyte characterization

**FIGURE 3:**
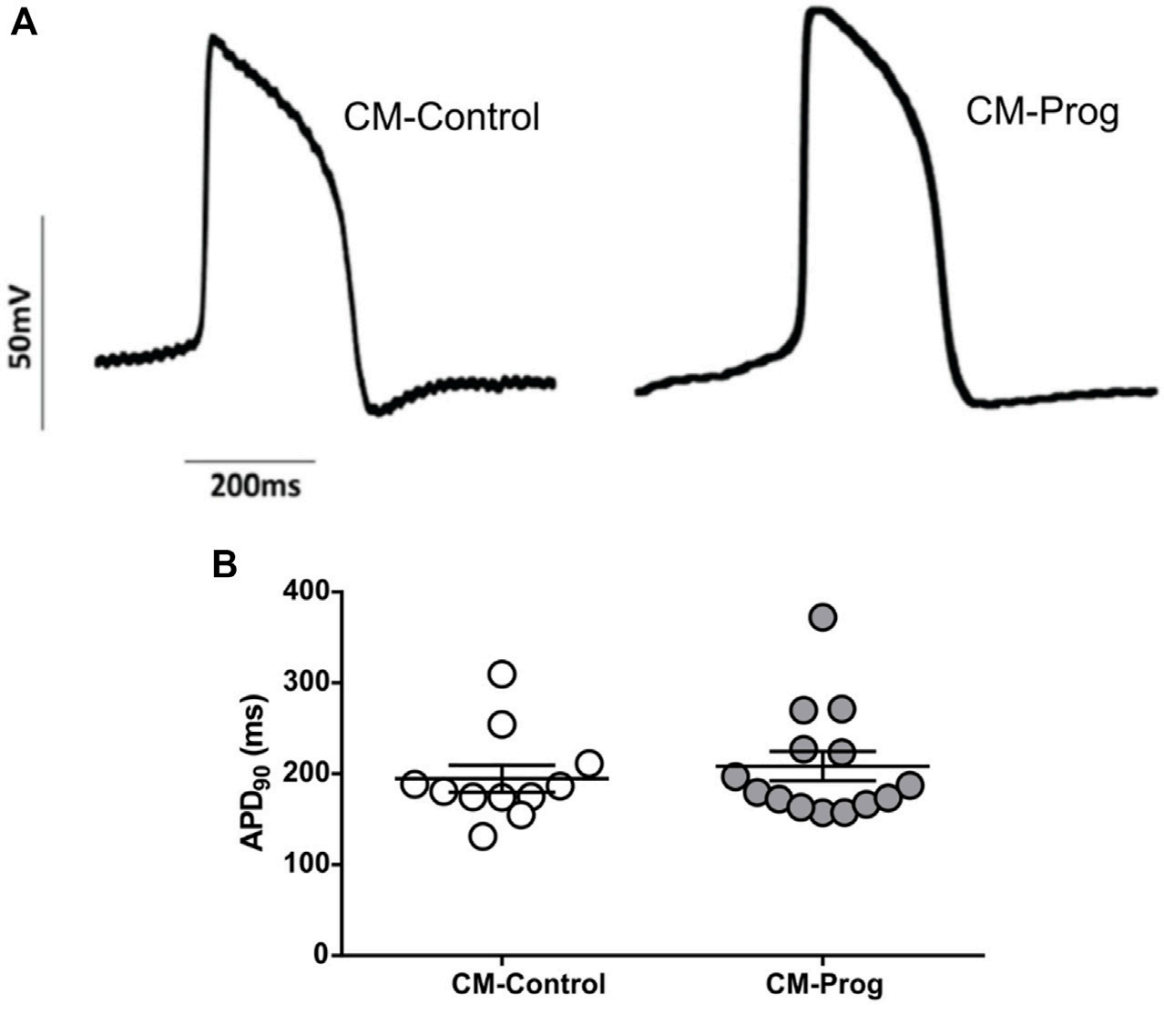
Electrophysiological characterization of iPSC-derived cardiomyocytes **(A)** Representative traces of action potential recordings in spontaneously contracting ventricular-like cardiomyocytes, from both control (CM-Control) and HGPS (CM-Prog) from three distinct cellular differentiations. **(B)** Action potential duration at 90% of repolarization (APD90). Scatter plot shows values from individual cells, where horizontal bars represent mean values with SEM. Each dot represents the action potential of a different cell and student’s t-test was applied (CM-Control *n* = 11; CM-Prog *n* = 14).

Cellular electrophysiology was analyzed in order to investigate whether the generated cardiomyocytes were functional. As demonstrated in Figure 3A, CM-Control and CM-Prog showed typical ventricle-like action potentials. Furthermore, we analyzed action potential duration at 90% (APD90) repolarization in spontaneously beating cardiomyocytes and found no difference between groups (Figure 3B). Furthermore, resting potential amplitude and maximal upstroke velocity were also similar in CM-Control and CM-Prog (Supplementary Table S1).

### 3.4 Cardiac mitochondrial morphologic and functional evaluation

**FIGURE 4:**
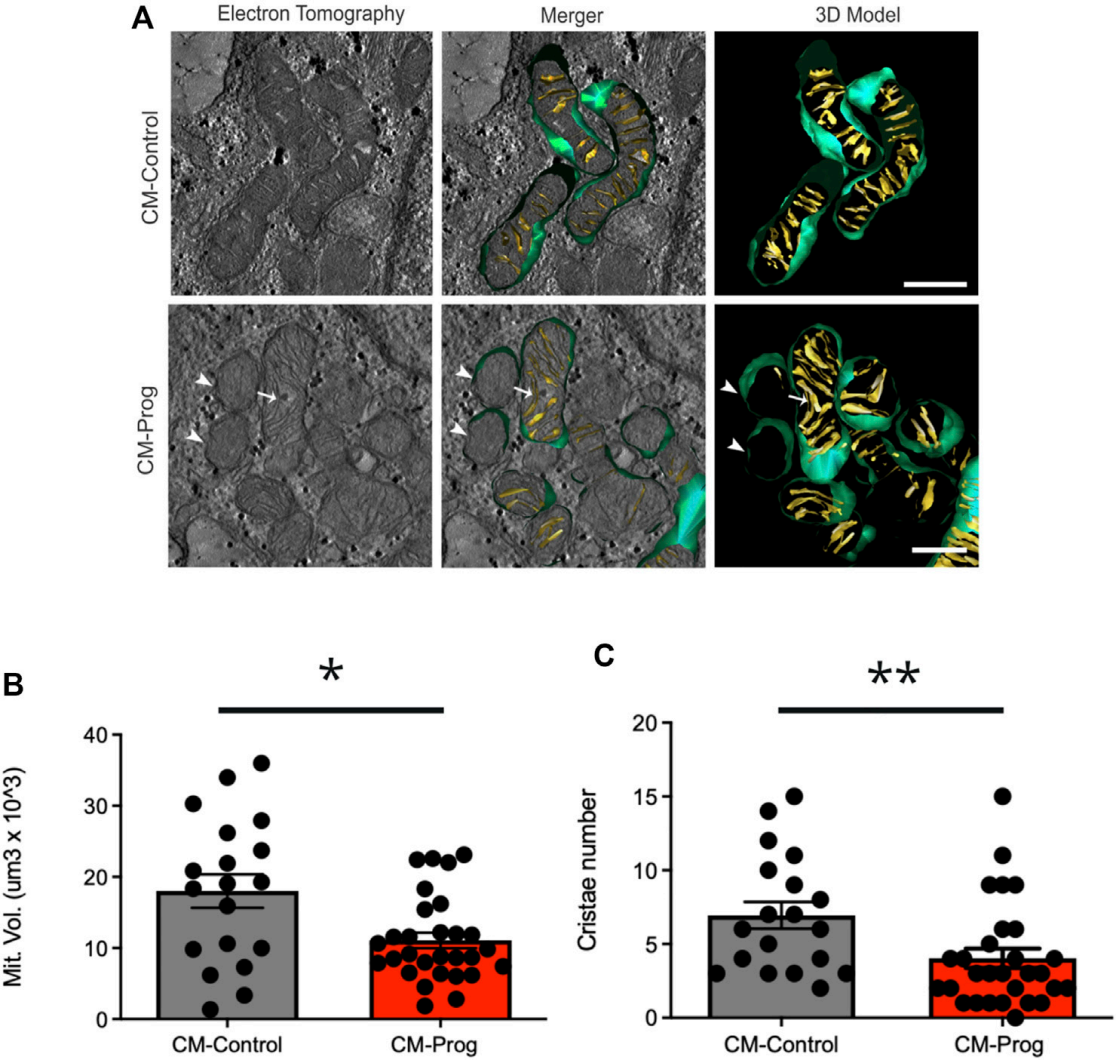
Electron tomography and mitochondrial 3D model in cardiomyocytes **(A)** Virtual z-slices obtained from tomograms, merged image and representative 3D model of control and HGPS cardiomyocytes. Mitochondria presenting an absence of cristae (arrowhead) or cristae running parallel to the main axis of the organelle (arrow) were observed in the CM-Prog condition. Scale bar: 300 nm. **(B)** Mitochondrial volume quantification. **(C)** Total number of mitochondrial cristae. Scatter plot shows values from individual mitochondria; horizontal bars represent mean values with SEM. * indicates *p* < 0.05 and **p < 0.01 between groups with student’s t-test (CM-Control *n* = 19; CM-Prog *n* = 29).

Mitochondrial alterations are one of the hallmarks of aging and HGPS. The structural organization of mitochondria in control and progeria iPSCs derived cardiomyocytes was assessed by electron tomography and 3D reconstruction. Results showed that CM-Prog mitochondria presented some abnormalities, such as the absence of cristae (Figure 4A, arrowhead) or cristae running parallel to the main axis of the organelle (Figure 4A, arrow). Three-dimensional morphometry showed that HGPS derived CM presented a decreased mitochondrial volume and a lower number of cristae/mitochondrion (Figure 4B,C).

**FIGURE 5:**
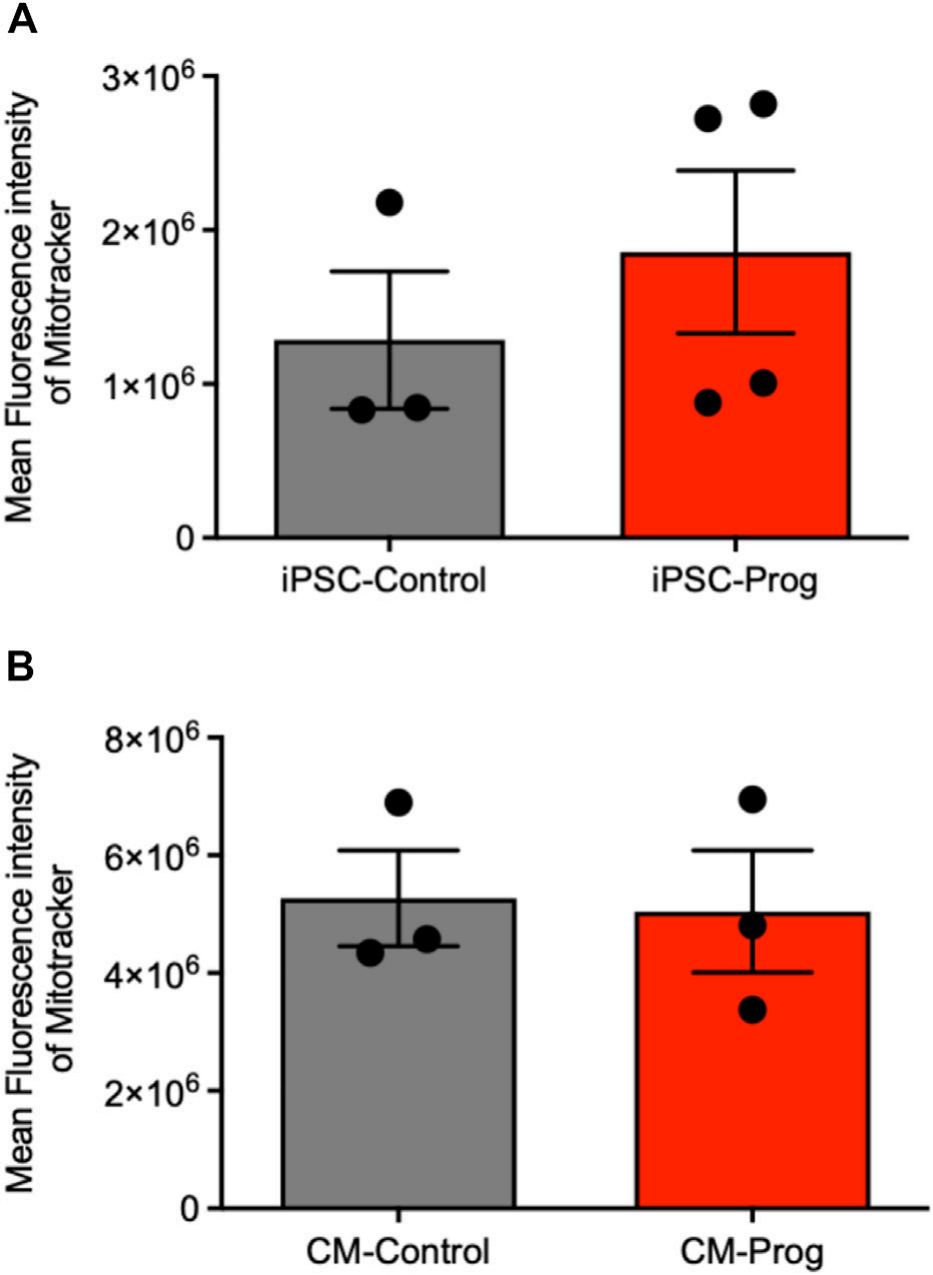
Mitochondrial content iPSCs and iPSCs derived cardiomyocytes. Mitochondrial content in iPSCs **(A)** and iPSCs derived cardiomyocytes **(B)** analyzed by Mitotracker Mean Fluorescence intensity. Columns represent mean values and bars SEM, and non-parametric Mann-Whitney test was applied (iPSC-Control *n* = 3; iPSC-Prog *n* = 4; CM-Control *n* = 3; CM-Prog *n* = 3).

**FIGURE 6:**
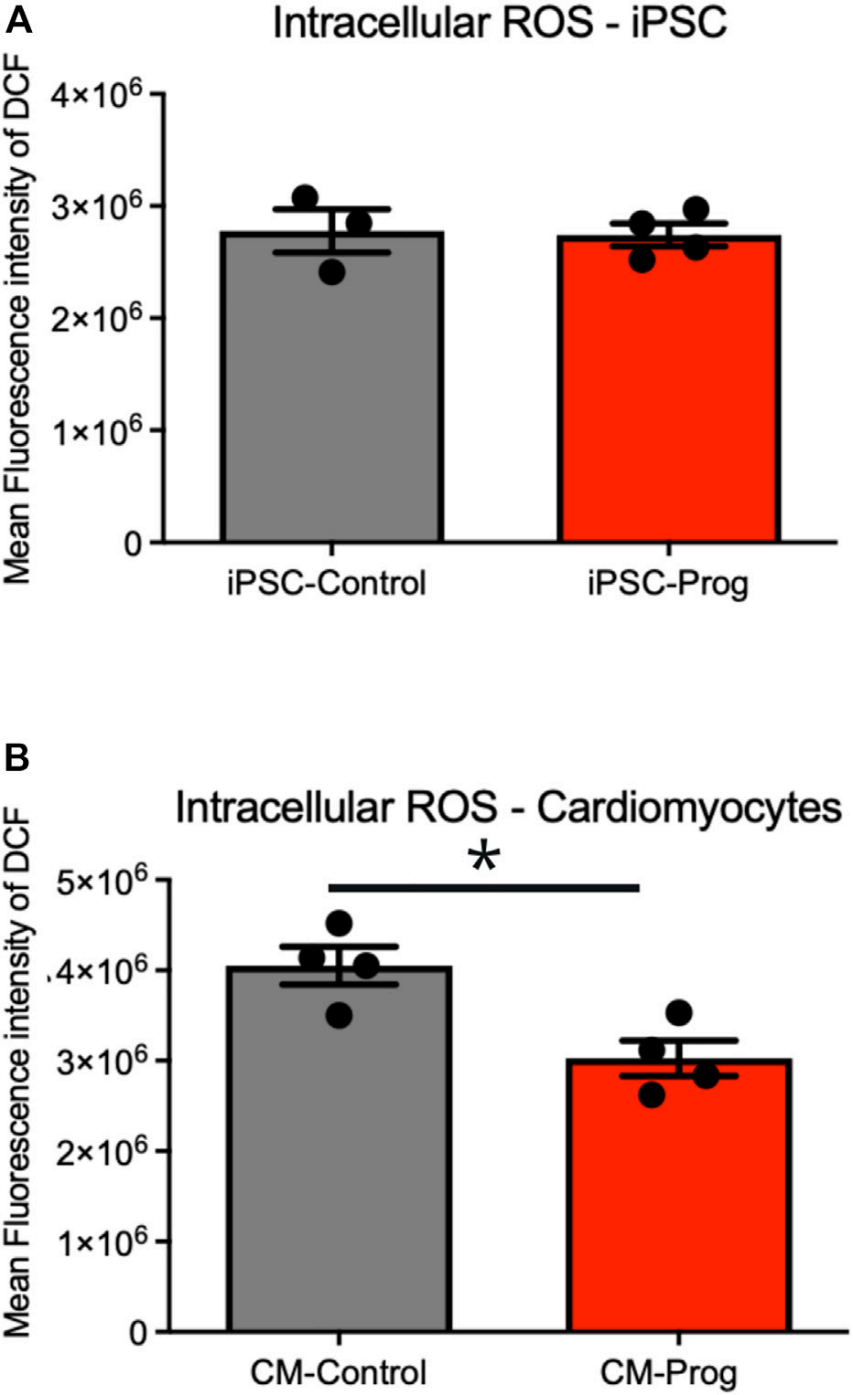
Intracellular generation of reactive oxidative species (ROS). Intracellular ROS production in iPSCs **(A)** and iPSCs derived cardiomyocytes **(B)** from cellular differentiations. Columns represent mean values and bars SEM. * indicates *p* < 0.05 between groups with non-parametric Mann-Whitney test was applied (iPSC-Control *n* = 3; iPSC-Prog *n* = 4; CM-Control *n* = 4; CM-Prog *n* = 4).

We next analyzed the mitochondrial content using Mitotracker in the iPSCs and the generated CM. As summarized in Figure 5A, there was no difference in mitochondrial content between control and HGPS iPSC. Furthermore, the generated CM presented similar mitochondrial content (Figure 5B), from three paired independent cellular differentiation protocols. We further analyzed ROS generation in the iPSCs and in the CM. As presented in Figure 6A, no difference was observed in ROS generation between the iPSCs groups. However, CM derived from HGPS iPSCs (CM-Prog) presented a significantly lower level of ROS production when compared to CM-Control (Figure 6B).

#### 3.5 Molecular alterations of the generated cardiomyocytes

**FIGURE 7:**
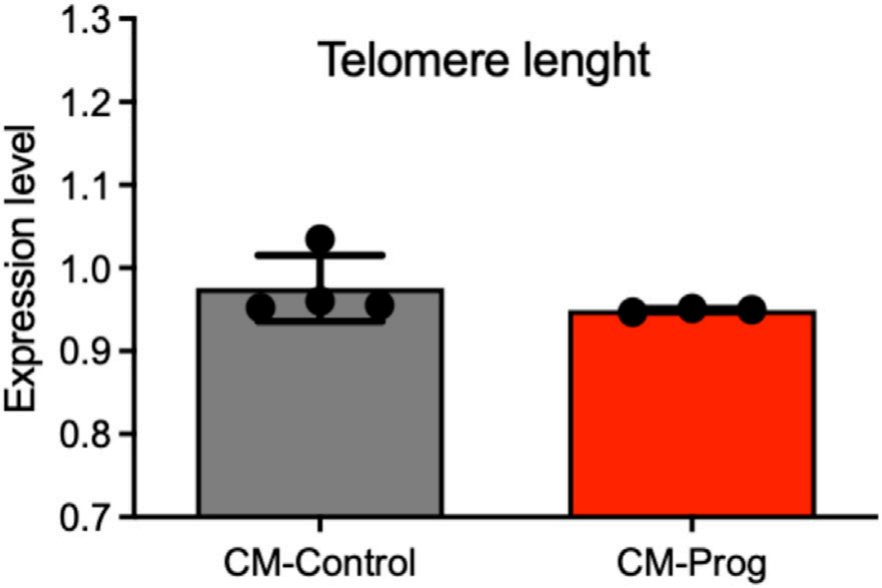
Telomere length in iPSC-derived cardiomyocytes. Telomere Length analysis in iPSC-derived cardiomyocytes performed by qPCR from distinct cellular differentiations. Columns represent mean values and bars SEM, and non-parametric Mann-Whitney test was applied (CM-Control *n* = 4; CM-Prog *n* = 3).

Telomere length is an important biomarker of aging biology. In this context, we investigated telomere length in CM from control and HGPS donors. As demonstrated in Figure 7, the CM presented normal telomere length.

Proteomic analysis of IPSCs derived cardiomyocytes.

**FIGURE 8:**
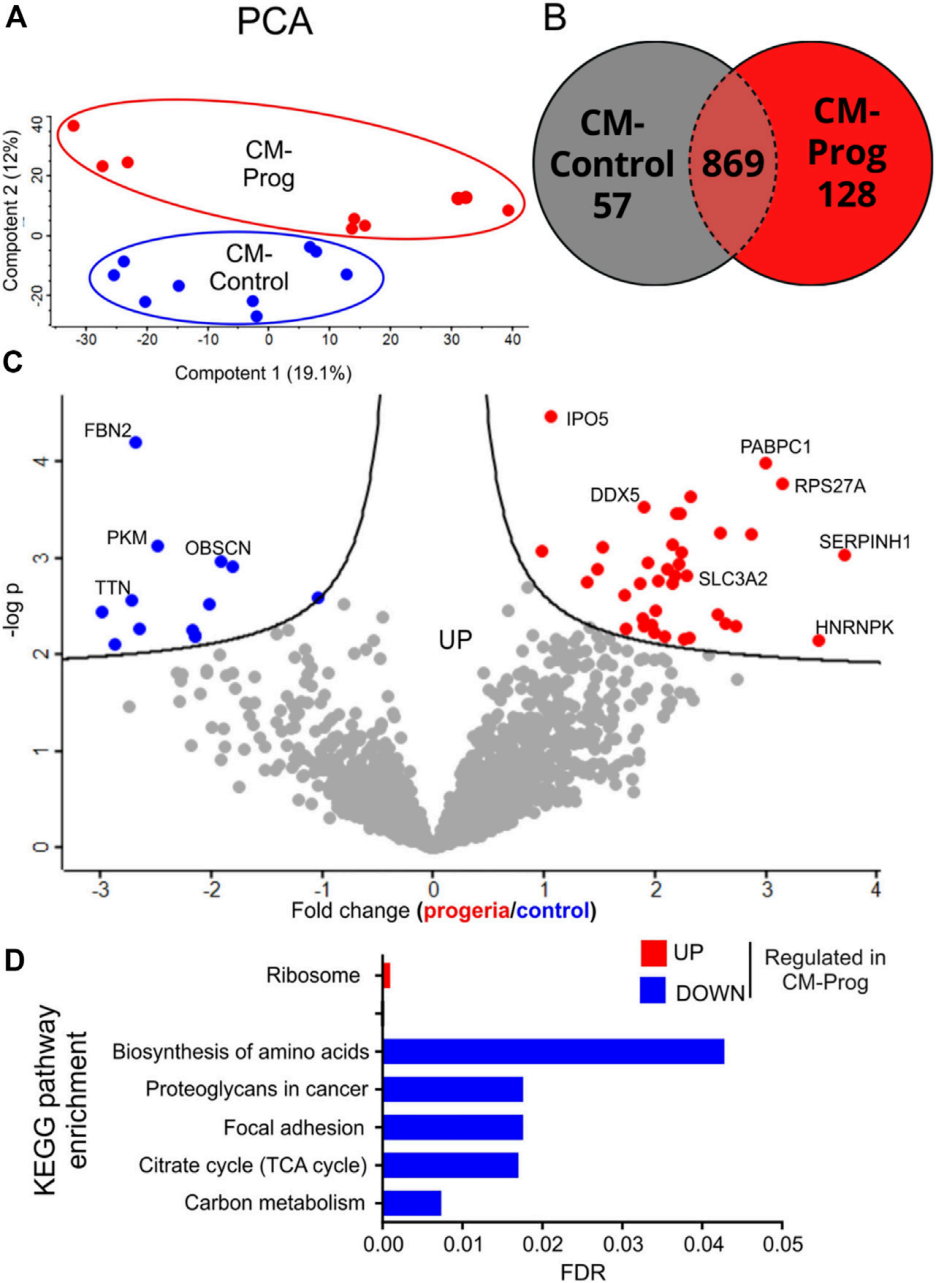
Proteomic analysis of Control and HGPS cardiomyocytes **(A)** Principal Component Analysis (PCA) of three distinct differentiations of iPS-derived cardiomyocytes from a Control and an HGPS patient run in triplicate. **(B)** Commonly and exclusively identified proteins in cardiomyocytes: a total of 1,054 proteins were identified; 869 proteins were expressed in both groups; 128 proteins were only expressed in Progeria and 57 proteins only in control. **(C)** Volcano plots showing differently expressed proteins: *p* values vs. the fold change in protein abundance between the two groups, where proteins above the lines in red are upregulated in Progeria and in blue are downregulated in Progeria) (FDR<0.1). **(D)** Gene Ontology Biological Process (KEGG Pathways) of differentially expressed proteins (CM-Control *n* = 3; CM-Prog *n* = 3; analyzed in triplicate).

To identify molecular patterns and networks altered by HGPS in cardiac cells, we performed a proteomic analysis based on liquid chromatography tandem mass spectrometry (LC-MS/MS) in a high-resolution system-based proteomic analysis. Principal Component Analysis (PCA) of three independent differentiation protocols with similar troponin T expression from a control and an HGPS patient run in triplicate shows methodological reproducibility and distinct group separation (Figure 8A). Commonly and exclusively identified proteins in iPSC-derived cardiomyocytes are summarized using Venn a diagram (Figure 8B), where a total of 1,054 proteins were identified; 869 proteins that were expressed in both groups; 128 proteins only expressed in HGPS and 57 proteins expressed only in Control (list of identified proteins is presented in Supplementary Table S2). Volcano plot analysis of the data shows differentially expressed proteins in iPS-derived cardiomyocytes from control and HGPS (Figure 8C), where proteins outside the significance lines are colored in red (upregulated in HGPS) or blue (downregulated in HGPS) (line represents FDR < 0.1). The list and statistical values of proteins identified with statistical differences between the two groups are presented in Supplementary Table S3. In order to better understand the biological role of the altered proteins, pathways analysis using Gene Ontology Biological Process of proteins differentially expressed (either down-regulated (blue) or up-regulated (red)) was performed as summarized in Figure 8D and in the Supplementary Table S4.

## 4 Discussion

Investigations into rare diseases such as HGPS are normally neglected due to high cost and low patient/sample availability (Collins, 2016), leading to poor understanding of disease pathophysiology, reduced treatment innovations and drug discovery (Chang et al., 2018) Furthermore, when these diseases affect tissues or organs that are difficult and risky to access, the issue of sample availability becomes even more critical. Therefore, the generation of functional cardiac cells derived from iPSCs from HGPS, a very rare disease where children present a high incidence of cardiovascular complications, opens novel ways to investigate the biological process of premature cardiac aging, discover new drugs (Mercola et al., 2013) and fulfill the promise of personalized medicine (Sharma et al., 2014). Herein we present, for the first time, a cellular and molecular functional analysis of cardiomyocytes derived from a Progeria Syndrome iPSCs cell line.

Critical complications in cardiac disease are often caused by electrical disturbances (Monnerat et al., 2016). Thus, in the present study, we analyzed the electrophysiological properties of the generated cardiomyocytes. No electrical abnormalities were observed in cardiomyocytes derived from HGPS iPSC. Interestingly, patients with HGPS display normal electrocardiograms throughout most of the course of the disease. Nevertheless, in the later stages of the disease, HGPS patients can present with QT interval prolongation (Merideth et al., 2008).

The generated cardiomyocytes derived from HGPS-iPSCs produced lower levels of ROS, however, ROS has been implicated in aging, and fibroblasts derived from HGPS patients have been shown to produce more ROS than control fibroblasts (Viteri et al., 2010; Mateos et al., 2015). We speculate that the diminished ROS production in cardiomyocytes derived from HGPS-iPSCs may be related to the lower mitochondrial function related to their morphological alteration, decreased number of cristae, and biochemical alterations found in the proteomics. Alterations found in mitochondria are compatible with altered metabolic function and capacity found in patients (Viteri et al., 2010; Gordon et al., 2014).

Besides mitochondrial alterations, the expression of key proteins is a determinant for energy generation and cellular homeostasis (Calvo and Mootha, 2010; Stefely et al., 2016). Using mass spectrometry, we were able to identify more than 1700 proteins in the cardiomyocytes derived from iPSC, many of them exclusively expressed in HGPS iPSC-derived cardiomyocytes or presenting significantly altered abundance. Interestingly, KEGG pathway analysis demonstrated that down-regulated proteins in HGPS cardiomyocytes are related to the TCA cycle, a key metabolic process for energy generation (Sheydina et al., 2011; López-Otín et al., 2013; Tocchi et al., 2015). In agreement with these findings, using a mouse model of HGPS, Rivera-Torres and coworkers demonstrated that metabolic alterations induced by HGPS might be related to a decreased mitochondrial oxidative capacity. In this study, the authors demonstrated that proteins necessary for mitochondrial oxidative phosphorylation are down-regulated in fibroblasts using a very sensitive mass spectrometry-based proteomics (Rivera-Torres et al., 2013). Furthermore, proteins related to the biosynthesis of amino acids were found to be down-regulated in our work. In this regard, frailty resulting from muscle loss is a major problem in the aged population. Besides, HGPS patients present low skeletal development that may be related to the growth abnormalities observed in HGPS children (Gordon et al., 2012).

Interestingly, previous publications demonstrated inhibition of PGC-1α function, a central regulator of mitochondrial biogenesis (Xiong et al., 2016), in HGPS fibroblasts. From the therapeutic point of view, drugs targeting this organelle might provide novel treatments. In this context, the treatment of fibroblasts from HGPS with metformin, a popular antidiabetic biguanide used for aging-related complications, is able to restore several metabolic and molecular alterations induced by premature aging, through AMPK activation, a key pathway also related to PGC-1α(Cantó and Auwerx, 2009; Monnerat-Cahli et al., 2014; Park and Shin, 2017).

Previous studies showed that the telomere is shortened in HGPS fibroblasts, but normal in hematopoietic cells (Decker et al., 2009). We observed no differences in telomere length between cardiomyocytes derived from healthy and HGPS donors. Telomere length shortening can differ between proliferative and minimally proliferative tissues, mainly during early life (Gardner et al., 2007; Daniali et al., 2013). Since iPSC-derived cells are known to be immature and cardiomyocytes are not very proliferative even in early developmental stages, the lack of a difference in telomere shortening between the control and HGPS cardiomyocytes is not surprising. To date, no study has measured telomere length in cardiomyocytes from HGPS patients.

Cellular stress is a major hallmark of cardiac diseases and aging. Cellular stress in cardiac cells can lead to promoting changes in several molecular pathways, leading to either activation or inactivation of key enzymes and other protein types. Therefore, a non-target proteomic approach is of major relevance for broad cellular investigation. Cell death, DNA damage, and abnormal calcium handling can be induced by cellular stress in cardiomyocytes, which increases the risk of cardiac diseases and premature aging (Yousefzadeh et al., 2021). In this context, our proteomics experiments found an increase of RPS27a in HPGS cardiomyocytes, a protein that plays an important role in DNA damage response regulated by p53 and was characterized as a cellular stress sensor (Nosrati et al., 2015). Furthermore, the heat shock protein 47, also known as SERPINH1, a protein expressed in the endoplasmic reticulum that is linked to stress response, was upregulated. Conversely, the OBSCN protein, from the obscurin proteins, was found to be decreased in the HGPS cardiomyocytes. Interestingly, different clinical studies have associated hypertrophic cardiomyopathies and a higher risk of sudden cardiac death with mutations in the OBSCN gene (Grogan and Kontrogianni-Konstantopoulos, 2019).

Our data indicate that premature cardiac aging is related to mitochondrial dysfunction, induced by morphological and biochemical alterations. Our study, however, has limitations. First, the use of a single iPSCs cell line from HGPS, and we are currently working on deriving more iPSCs lines from different patients. Second, the cardiomyocytes derived from iPSCs are known to be immature and may not present the same physiological properties as adult cardiomyocytes. Third, we used an iPSCs control cell line from a completely non-related patient line, an isogenic control with a similar genetic background might influence the experimental results found in the present study. Nonetheless, significant differences in cardiomyocyte protein expression, metabolic pathways, and nuclear and mitochondrial morphology were evident in cardiomyocytes derived from HGPS iPSCs when compared to those derived from a normal donor. We believe that these findings will induce further research using iPSCs derived cells from extremely rare diseases.

## 5 Conclusion

Our study modeled cardiac premature aging using induced pluripotent stem cells from a patient with Progeria Syndrome, a very rare disease that affects children. Taken together, our data show distinct mitochondrial properties in cardiomyocytes derived from HGPS iPSC, including morphological and biochemical alterations. Our findings create novel insights for premature cardiac aging and a platform that may be further explored for *in vitro* drug screening, in the search for new drugs to ameliorate the devastating course of HGPS.

## Data Availability

All the data used to support the findings of this study are included within the article and supplementary material files. All the proteomics data are available online (ProteomeXchange Consortium via the PRIDE (Perez-Riverol et al., 2022) partner repository with the dataset identifier PXD036557).

## References

[B1] Atchison L. Abutaleb N. O. Snyder-Mounts E. Gete Y. Ladha A. Ribar T. (2020). IPSC-derived endothelial cells affect vascular function in a tissue-engineered blood vessel model of Hutchinson-Gilford progeria syndrome. Stem Cell. Rep. 14, 325–337. 10.1016/j.stemcr.2020.01.005 PMC7013250 32032552

[B2] Barzilai N. Huffman D. M. Muzumdar R. H. Bartke A. (2012). The critical role of metabolic pathways in aging. Diabetes 61, 1315–1322. 10.2337/db11-1300 22618766 PMC3357299

[B3] Bonello-Palot N. Simoncini S. Robert S. Bourgeois P. Sabatier F. Levy N. (2014). Prelamin A accumulation in endothelial cells induces premature senescence and functional impairment. Atherosclerosis 237, 45–52. 10.1016/j.atherosclerosis.2014.08.036 25200614

[B4] Bonomini F. Rodella L. F. Rezzani R. (2015). Metabolic syndrome, aging and involvement of oxidative stress. Aging Dis. 6, 109–120. 10.14336/AD.2014.0305 25821639 PMC4365955

[B5] Calvo S. E. Mootha V. K. (2010). The mitochondrial proteome and human disease. Annu. Rev. Genomics Hum. Genet. 11, 25–44. 10.1146/annurev-genom-082509-141720 20690818 PMC4397899

[B6] Cantó C. Auwerx J. (2009). PGC-1alpha, SIRT1 and AMPK, an energy sensing network that controls energy expenditure. Curr. Opin. Lipidol. 20, 98–105. 10.1097/MOL.0b013e328328d0a4 19276888 PMC3627054

[B7] Capell B. C. Collins F. S. Nabel E. G. (2007). Mechanisms of cardiovascular disease in accelerated aging syndromes. Circ. Res. 101, 13–26. 10.1161/CIRCRESAHA.107.153692 17615378

[B8] Capell B. C. Olive M. Erdos M. R. Cao K. Faddah D. A. Tavarez U. L. (2008). A farnesyltransferase inhibitor prevents both the onset and late progression of cardiovascular disease in a progeria mouse model. Proc. Natl. Acad. Sci. U. S. A. 105, 15902–15907. 10.1073/pnas.0807840105 18838683 PMC2562418

[B9] Chang C. Y. Ting H. C. Liu C. A. Su H. L. Chiou T. W. Harn H. J. (2018). Induced Pluripotent Stem Cells: A Powerful Neurodegenerative Disease Modeling Tool for Mechanism Study and Drug Discovery, 11, 1588–1602. 10.1177/0963689718775406 Cell. Transpl. PMC6299199 29890847

[B10] Chen F. Liu Y. Wong N. K. Xiao J. So K. F. (2017). Oxidative stress in stem cell aging. Cell. Transpl. 26, 1483–1495. 10.1177/0963689717735407 PMC5680960 29113471

[B11] Collins F. S. (2016). Seeking a cure for one of the rarest diseases: Progeria. Circulation 134, 126–129. 10.1161/CIRCULATIONAHA.116.022965 27400897 PMC5101939

[B12] Daniali L. Benetos A. Susser E. Kark J. D. Labat C. Kimura M. (2013). Telomeres shorten at equivalent rates in somatic tissues of adults. Nat. Commun. 4, 1597. 10.1038/ncomms2602 23511462 PMC3615479

[B13] Decker M. L. Chavez E. Vulto I. Lansdorp P. M. (2009). Telomere length in Hutchinson-Gilford progeria syndrome. Mech. Ageing Dev. 130, 377–383. 10.1016/j.mad.2009.03.001 19428457

[B14] Gardner J. P. Kimura M. Chai W. Durrani J. F. Tchakmakjian L. Cao X. (2007). Telomere dynamics in macaques and humans. J. Gerontol. A Biol. Sci. Med. Sci. 62, 367–374. 10.1093/gerona/62.4.367 17452729

[B15] Gordon L. B. Kleinman M. E. Miller D. T. Neuberg D. S. Giobbie-Hurder A. Gerhard-Herman M. (2012). Clinical trial of a farnesyltransferase inhibitor in children with Hutchinson-Gilford progeria syndrome. Proc. Natl. Acad. Sci. U. S. A. 109, 16666–16671. 10.1073/pnas.1202529109 23012407 PMC3478615

[B16] Gordon L. B. Rothman F. G. López-Otín C. Misteli T. (2014). Progeria: A paradigm for translational medicine. Cell. 156, 400–407. 10.1016/j.cell.2013.12.028 24485450 PMC6318797

[B17] Gordon L. B. Shappell H. Massaro J. D’Agostino R. B. Brazier J. Campbell S. E. (2018). Association of lonafarnib treatment vs No treatment with mortality rate in patients with hutchinson-gilford progeria syndrome. JAMA 319, 1687–1695. 10.1001/jama.2018.3264 29710166 PMC5933395

[B18] Grogan A. Kontrogianni-Konstantopoulos A. (2019). Unraveling obscurins in heart disease. Pflugers Arch. 471, 735–743. 10.1007/s00424-018-2191-3 30099631 PMC6397709

[B19] Gutierrez-Rodrigues F. Santana-Lemos B. A. Scheucher P. S. Alves-Paiva R. M. Calado R. T. (2014). Direct comparison of flow-FISH and qPCR as diagnostic tests for telomere length measurement in humans. PLoS One 9, e113747. 10.1371/journal.pone.0113747 25409313 PMC4237503

[B20] Hamczyk M. R. Villa-Bellosta R. Gonzalo P. Andrés-Manzano M. J. Nogales P. Bentzon J. F. (2018). Vascular smooth muscle-specific progerin expression accelerates atherosclerosis and death in a mouse model of hutchinson-gilford progeria syndrome. Circulation 138, 266–282. 10.1161/CIRCULATIONAHA.117.030856 29490993 PMC6075893

[B21] Harley C. B. Futcher A. B. Greider C. W. (1990). Telomeres shorten during ageing of human fibroblasts. Nature 345, 458–460. 10.1038/345458a0 2342578

[B22] Hennekam R. C. (2006). Hutchinson-gilford progeria syndrome: Review of the phenotype. Am. J. Med. Genet. A 140, 2603–2624. 10.1002/ajmg.a.31346 16838330

[B23] José V. S. S. Monnerat G. Guerra B. Paredes B. D. Kasai-Brunswick T. H. Carvalho A. C. C. (2017). Bone-Marrow-derived mesenchymal stromal cells (MSC) from diabetic and nondiabetic rats have similar therapeutic potentials. Arq. Bras. Cardiol. 109, 579–589. 10.5935/abc.20170176 29364350 PMC5783439

[B24] Kenyon C. J. (2010). The genetics of ageing. Nature 464, 504–512. 10.1038/nature08980 20336132

[B25] Liu G. H. Barkho B. Z. Ruiz S. Diep D. Qu J. Yang S. L. (2011). Recapitulation of premature ageing with iPSCs from Hutchinson-Gilford progeria syndrome. Nature 472, 221–225. 10.1038/nature09879 21346760 PMC3088088

[B26] López-Otín C. Blasco M. A. Partridge L. Serrano M. Kroemer G. (2013). The hallmarks of aging. Cell. 153, 1194–1217. 10.1016/j.cell.2013.05.039 23746838 PMC3836174

[B27] Ma S. Yim S. H. Lee S. G. Kim E. B. Lee S. R. Chang K. T. (2015). Organization of the mammalian metabolome according to organ function, lineage specialization, and longevity. Cell. Metab. 22, 332–343. 10.1016/j.cmet.2015.07.005 26244935 PMC4758382

[B28] Mateos J. Landeira-Abia A. Fafián-Labora J. A. Fernández-Pernas P. Lesende-Rodríguez I. Fernández-Puente P. (2015). iTRAQ-based analysis of progerin expression reveals mitochondrial dysfunction, reactive oxygen species accumulation and altered proteostasis. Stem Cell. Res. Ther. 6, 119. 10.1186/s13287-015-0110-5 26066325 PMC4487579

[B29] Mercola M. Colas A. Willems E. (2013). Induced pluripotent stem cells in cardiovascular drug discovery. Circ. Res. 112, 534–548. 10.1161/CIRCRESAHA.111.250266 23371902 PMC3706265

[B30] Merideth M. A. Gordon L. B. Clauss S. Sachdev V. Smith A. C. Perry M. B. (2008). Phenotype and course of Hutchinson-Gilford progeria syndrome. N. Engl. J. Med. 358, 592–604. 10.1056/NEJMoa0706898 18256394 PMC2940940

[B31] Mesquita F. C. Kasai-Brunswick T. H. Gubert F. e. M. Borgonovo T. Silva-dos-Santos D. de Araújo D. S. (2015). Generation of human iPS cell line ihFib3.2 from dermal fibroblasts. Stem Cell. Res. 15, 445–448. 10.1016/j.scr.2015.09.001 26413783

[B32] Monnerat G. Alarcón M. L. Vasconcellos L. R. Hochman-Mendez C. Brasil G. Bassani R. A. (2016). Macrophage-dependent IL-1β production induces cardiac arrhythmias in diabetic mice. Nat. Commun. 7, 13344. 10.1038/ncomms13344 27882934 PMC5123037

[B33] Monnerat G. Brunswick T. Asensi K. Santos D. Andrade R. Mesquita F. (2018a). Abstract 503: Modeling premature cardiac aging by induced pluripotent stem cell from a patient with Hutchinson-Gilford Progeria Syndrome. Circ. Res. 123. 10.1161/res.123.suppl_1.503

[B34] Monnerat G. Seara F. A. C. Evaristo J. A. M. Carneiro G. Evaristo G. P. C. Domont G. (2018b). Aging-related compensated hypogonadism: Role of metabolomic analysis in physiopathological and therapeutic evaluation. J. Steroid Biochem. Mol. Biol. 183, 39–50. 10.1016/j.jsbmb.2018.05.005 29920416

[B35] Monnerat-Cahli G. Trentin-Sonoda M. Guerra B. Manso G. Ferreira A. C. Silva D. L. (2014). Bone marrow mesenchymal stromal cells rescue cardiac function in streptozotocin-induced diabetic rats. Int. J. Cardiol. 171, 199–208. 10.1016/j.ijcard.2013.12.013 24374203

[B36] Nogueira F. C. S. Farias A. R. B. Teixeira F. M. Domont G. B. Campos F. A. P. (2018). Common features between the proteomes of floral and extrafloral nectar from the Castor plant (ricinus communis) and the proteomes of exudates from carnivorous plants. Front. Plant Sci. 9, 549. 10.3389/fpls.2018.00549 29755492 PMC5934526

[B37] North B. J. Sinclair D. A. (2012). The intersection between aging and cardiovascular disease. Circ. Res. 110, 1097–1108. 10.1161/CIRCRESAHA.111.246876 22499900 PMC3366686

[B38] Nosrati N. Kapoor N. R. Kumar V. (2015). DNA damage stress induces the expression of ribosomal protein S27a gene in a p53-dependent manner. Gene 559, 44–51. 10.1016/j.gene.2015.01.014 25592822

[B39] Olive M. Harten I. Mitchell R. Beers J. K. Djabali K. Cao K. (2010). Cardiovascular pathology in hutchinson-gilford progeria: Correlation with the vascular pathology of aging. Arterioscler. Thromb. Vasc. Biol. 30, 2301–2309. 10.1161/ATVBAHA.110.209460 20798379 PMC2965471

[B40] Park S. K. Shin O. S. (2017). Metformin alleviates ageing cellular phenotypes in Hutchinson-Gilford progeria syndrome dermal fibroblasts. Exp. Dermatol. 26, 889–895. 10.1111/exd.13323 28192606

[B41] Perez-Riverol Y. Bai J. Bandla C. García-Seisdedos D. Hewapathirana S. Kamatchinathan S. (2022). The PRIDE database resources in 2022: A hub for mass spectrometry-based proteomics evidences. Nucleic Acids Res. 50, D543–D552. 10.1093/nar/gkab1038 34723319 PMC8728295

[B42] Rando T. A. (2006). Stem cells, ageing and the quest for immortality. Nature 441, 1080–1086. 10.1038/nature04958 16810243

[B43] Rivera-Torres J. Acín-Perez R. Cabezas-Sánchez P. Osorio F. G. Gonzalez-Gómez C. Megias D. (2013). Identification of mitochondrial dysfunction in Hutchinson-Gilford progeria syndrome through use of stable isotope labeling with amino acids in cell culture. J. Proteomics 91, 466–477. 10.1016/j.jprot.2013.08.008 23969228

[B44] Roumeliotis T. I. Williams S. P. Gonçalves E. Alsinet C. Del Castillo Velasco-Herrera M. Aben N. (2017). Genomic determinants of protein abundance variation in colorectal cancer cells. Cell. Rep. 20, 2201–2214. 10.1016/j.celrep.2017.08.010 28854368 PMC5583477

[B45] Sharma A. Marceau C. Hamaguchi R. Burridge P. W. Rajarajan K. Churko J. M. (2014). Human induced pluripotent stem cell-derived cardiomyocytes as an *in vitro* model for coxsackievirus B3-induced myocarditis and antiviral drug screening platform. Circ. Res. 115, 556–566. 10.1161/CIRCRESAHA.115.303810 25015077 PMC4149868

[B46] Sheydina A. Riordon D. R. Boheler K. R. (2011). Molecular mechanisms of cardiomyocyte aging. Clin. Sci. 121, 315–329. 10.1042/CS20110115 PMC5928524 21699498

[B47] Silva Dos Santos D. Brasil G. V. Ramos I. P. R. Mesquita F. C. P. Kasai-Brunswick T. H. Christie M. L. A. (2018). Embryonic stem cell-derived cardiomyocytes for the treatment of doxorubicin-induced cardiomyopathy. Stem Cell. Res. Ther. 9, 30. 10.1186/s13287-018-0788-2 29402309 PMC5799903

[B48] Stefely J. A. Kwiecien N. W. Freiberger E. C. Richards A. L. Jochem A. Rush M. J. P. (2016). Mitochondrial protein functions elucidated by multi-omic mass spectrometry profiling. Nat. Biotechnol. 34, 1191–1197. 10.1038/nbt.3683 27669165 PMC5101133

[B49] Szklarczyk D. Morris J. H. Cook H. Kuhn M. Wyder S. Simonovic M. (2017). The STRING database in 2017: Quality-controlled protein-protein association networks, made broadly accessible. Nucleic Acids Res. 45, D362–D368. 10.1093/nar/gkw937 27924014 PMC5210637

[B50] Takahashi K. Tanabe K. Ohnuki M. Narita M. Ichisaka T. Tomoda K. (2007). Induction of pluripotent stem cells from adult human fibroblasts by defined factors. Cell. 131, 861–872. 10.1016/j.cell.2007.11.019 18035408

[B51] Tocchi A. Quarles E. K. Basisty N. Gitari L. Rabinovitch P. S. (2015). Mitochondrial dysfunction in cardiac aging. Biochim. Biophys. Acta 1847, 1424–1433. 10.1016/j.bbabio.2015.07.009 26191650 PMC4575872

[B52] Tyanova S. Temu T. Sinitcyn P. Carlson A. Hein M. Y. Geiger T. (2016). The Perseus computational platform for comprehensive analysis of (prote)omics data. Nat. Methods 13, 731–740. 10.1038/nmeth.3901 27348712

[B53] Viteri G. Chung Y. W. Stadtman E. R. (2010). Effect of progerin on the accumulation of oxidized proteins in fibroblasts from Hutchinson Gilford progeria patients. Mech. Ageing Dev. 131, 2–8. 10.1016/j.mad.2009.11.006 19958786 PMC2837844

[B54] Wendt C. Rachid R. de Souza W. Miranda K. (2016). Electron tomography characterization of hemoglobin uptake in Plasmodium chabaudi reveals a stage-dependent mechanism for food vacuole morphogenesis. J. Struct. Biol. 194, 171–179. 10.1016/j.jsb.2016.02.014 26882843

[B55] Xiong Z. M. Choi J. Y. Wang K. Zhang H. Tariq Z. Wu D. (2016). Methylene blue alleviates nuclear and mitochondrial abnormalities in progeria. Aging Cell. 15, 279–290. 10.1111/acel.12434 26663466 PMC4783354

[B56] Yousefzadeh M. Henpita C. Vyas R. Soto-Palma C. Robbins P. Niedernhofer L. (2021). DNA damage-how and why we age? Elife 10, e62852. 10.7554/eLife.62852 33512317 PMC7846274

[B57] Zhang J. Wilson G. F. Soerens A. G. Koonce C. H. Yu J. Palecek S. P. (2009). Functional cardiomyocytes derived from human induced pluripotent stem cells. Circ. Res. 104, e30–e41. 10.1161/CIRCRESAHA.108.192237 19213953 PMC2741334

